# Status and Prospects of ZnO-Based Resistive Switching Memory Devices

**DOI:** 10.1186/s11671-016-1570-y

**Published:** 2016-08-19

**Authors:** Firman Mangasa Simanjuntak, Debashis Panda, Kung-Hwa Wei, Tseung-Yuen Tseng

**Affiliations:** 1 Department of Materials Science and Engineering, National Chiao Tung University, Hsinchu, 30010 Taiwan; 2 Department of Electronics Engineering, National Institute of Science and Technology, Berhampur, Odisha 761008 India; 3 Department of Electronics Engineering and Institute of Electronics, National Chiao Tung University, Hsinchu, 30010 Taiwan

**Keywords:** Resistive switching, Resistive memory, RRAM, Memristor, ZnO, Nonvolatile memory

## Abstract

In the advancement of the semiconductor device technology, ZnO could be a prospective alternative than the other metal oxides for its versatility and huge applications in different aspects. In this review, a thorough overview on ZnO for the application of resistive switching memory (RRAM) devices has been conducted. Various efforts that have been made to investigate and modulate the switching characteristics of ZnO-based switching memory devices are discussed. The use of ZnO layer in different structure, the different types of filament formation, and the different types of switching including complementary switching are reported. By considering the huge interest of transparent devices, this review gives the concrete overview of the present status and prospects of transparent RRAM devices based on ZnO. ZnO-based RRAM can be used for flexible memory devices, which is also covered here. Another challenge in ZnO-based RRAM is that the realization of ultra-thin and low power devices. Nevertheless, ZnO not only offers decent memory properties but also has a unique potential to be used as multifunctional nonvolatile memory devices. The impact of electrode materials, metal doping, stack structures, transparency, and flexibility on resistive switching properties and switching parameters of ZnO-based resistive switching memory devices are briefly compared. This review also covers the different nanostructured-based emerging resistive switching memory devices for low power scalable devices. It may give a valuable insight on developing ZnO-based RRAM and also should encourage researchers to overcome the challenges.

## Review

### Introduction

Semiconductor memory is an indispensable component and backbone of all modern electronic devices. All recognizable computing platforms ranging from hand-held devices to large super-computer storage systems are used for storing data, either temporarily or permanently, as per their requirement [1]. Based on storing data volatility, memories are basically classified into two categories, (i) volatile memory and (ii) nonvolatile memory. In a volatile memory, the stored data is lost immediately after the power is turned off whereas nonvolatile memory (NVM) is capable to retain the stored data for a long time even after the power is off. Demands on NVMs are increasing extensively, due to the huge popularity of consumer electronics and portable gadgets, such as smart phone, memory card, and USB storage devices, where NVM is one of the basic component [2–5].

Over the last few decades, a variety of NVM devices such as flash memory, resistive random access memory (RRAM), phase change memory (PCM), ferroelectric memory (FeRAM), and magnetic random access memory (MRAM) have emerged, though each has some technical limits, such as scalability, retention, switching power, and reliability aspects [2, 5–33]. Among them, resistive switching memory devices are expected to be one of the promising candidates for future nanoscale memories [34–46].

#### RRAM Technology

In 1971 [47, 48], memristor (memory-resistor), later also called as resistive switching memory [31, 32], is firstly introduced and theorized as the fourth classical circuit elements by Chua. The element was realized in the form of active circuit which then behaves like a nonlinear resistor with memory [47]. A few years before the introduction of the new element, nonlinear resistance changes had been observed in various metal oxides [49]. Gibbons and Beadle, in 1964 [50], proposed the existence of conducting filament (CF) to control the resistance changes in Ag/NiO/Ni device. However, the origin of such conduction is not explored until Simmons and Verderber in 1967 [51] suggested that the conduction of reversible switching in Au/SiO/Al device was originated from the conduction electrons travel by tunnelling between sites provided by Au ions injected from Au electrode. These findings may lead to the development of RRAM applications. Nowadays, the role of CF is acknowledged to be as a “circuit breaker” that determine the principle of the switching itself [37]. Much efforts has been conducted to modulate its shape, size, and number and to understand the mechanism that define the switching behavior [52–56].

#### Advantages of ZnO for RRAM Applications

**Fig. 1:**
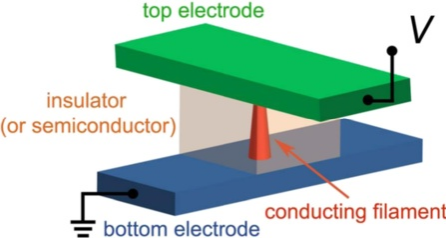
Schematic of conductor/insulator (or semiconductor)/conductor sandwich structure [43]

Resistive random access memory have been developed in various structures, such as sandwich [52–56], planar [57, 58], laterally bridge [59], single nanorod/nanowire [60–75], nanobelt [76], and nanoisland [77, 78]. Nevertheless, the basic RRAM structure should consist of two opposite electrodes and a storage material, as depicted in Fig. 1. Organic [79], inorganic [44,] or hybrid [80] insulating materials can be used as a storage material for the RRAM applications. Among them, inorganic materials, metal oxides, gained huge interest for the use as storage materials due to its wide range of electrical properties [39]. Among numerous metal oxides, ZnO has advantageous properties such as low cost, wide and direct band gap of ~3.3 eV, low synthetic temperature, controllable electrical behavior, chemically stable, electrochemical activity, biocompatible, and environmental friendly [81, 82]. ZnO can be grown with wide variety of morphologies [81, 82], such as nanowires, nanorods, terapods, nanoribbons/belts, hierarchical, bridge-/nail-like, tubular, nanosheets, nanopropeller, nanohelixes, and nanorings which may open the opportunity to fabricate various one-dimensional RRAM structures*.* Due to its exceptional advantages and various morphologies, ZnO has been also considered as a promising candidate in broad practical applications [81, 82], such as piezoelectric transducers, bio sensors, chemical and gas sensors, optical waveguides, photo detector, photovoltaics, surface acoustic wave devices, varistors, transparent conductive oxides, spin functional devices, and UV-light emitters. These wide applications may open the possibility to design nonvolatile resistive switching memories with multifunctional features which will be discussed later.

#### Switching Mechanism in Oxide-Based RRAM

**Fig. 2:**
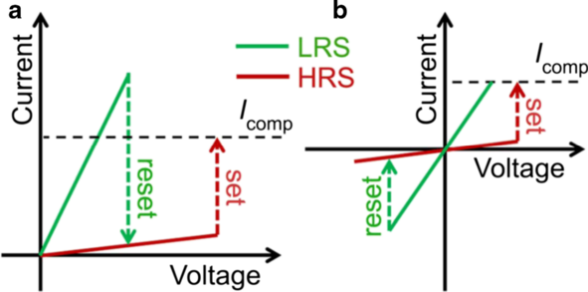
Schematic I-V curves of **a** unipolar and **b** bipolar switching. I_comp_ denotes the compliance current, which is adopted during set process to prevent permanent breakdown [43]

Computer data are read in the sense of binary code “1” and “0.” Accordingly, data stored in resistive memory devices are differentiated by its resistance state, so called “low resistance state (LRS)” or “ON” and “high resistance state (HRS)” or “OFF” states. These states can be switched reversely using electric stimulus. The switching process from HRS to LRS and LRS to HRS are named as set and reset, respectively. Current compliance (I_comp_) is normally applied to prevent hard breakdown during set. Resistive memory operates under either unipolar or bipolar operation mode. In unipolar mode, depicted in Fig. 2a, set and reset processes occur in the same bias polarity. Conversely, in bipolar mode, opposite bias polarities are required to set and reset a device, as depicted in Fig. 2b. These modes are dependent on device structure [44, 45, 83] and electrical operation setup [31, 84]. However, coexistence of bipolar and unipolar in the same device was also reported [85–88]. Nevertheless, general understanding on unipolar and bipolar modes can be concluded upon the factors that trigger the reset process. In unipolar, Joule heating is the main driving force to rupture a CF during reset, whereas in bipolar, dissolution of CF is due to the migrating charged species, yet Joule heating still contributes to accelerate the migration [42, 45].

Generally, based on the chemical effects involved in the switching process, RRAM can be classified as electrochemical metallization memory (ECM) and valence change memory (VCM) [44]. ECM, also known as conductive bridge CBRAM, relies on an electrochemically active metal electrode [42] such as Ag, Cu, or Ni, to form metal cation-based CF. On the other hand, CF in VCM cell is composed of oxygen vacancies defects, instead of metal atoms, due to anion migration within the storage material itself [31]. This CF size in the range of 20–30 nm strongly depends upon the amount of current flowed during forming and set [45, 89].

In filamentary model, the set current mainly flows through the CF [46]. The filament size is considerably smaller than electrode area that leads to localized conduction effect; thus, LRS is independent on electrode size [46, 86, 90]. Apart from the filamentary model, homogeneous interface-type model was also proposed in switching mechanism of VCM cell [43]. In homogeneous interface-type model, both set and reset current flow homogenously over the entire electrode area; thus, LRS and HRS are proportional to the electrode area [44]. The conduction is determined by the field-induced change of the Schottky barrier height at the electrode/storage material interface [42, 44]. Interface-type device can be designed by sandwiching the storage material with Ohmic and Schottky contacts [43, 91] or modulating oxide/oxide interface in multilayer device [83].

**Fig. 3:**
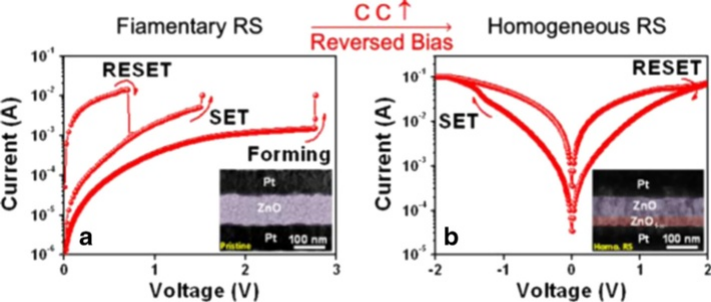
Transformation of filamentary into homogeneous resistive switching by applying reversed bias with high (I_comp_) in Pt/ZnO/Pt device. The insets in the left and right figures are TEM images of the device before and after transformation, respectively [84]

The filamentary switching can be transformed into homogeneous switching [84] by modulating the measurement parameters. Figure 3 shows the transformation of filamentary into homogeneous switching in a typical Pt/ZnO/Pt device [84]. The transformation was conducted by introducing a reverse sweep bias with high I_comp_ after initiating unipolar switch leading to the formation of oxygen-defective region near the bottom electrode, as depicted in the inset of Fig. 3b [84]. This region can also be modulated simply by applying various I_comp_ or reset voltage (V_reset_); hence, multilevel characteristic was observed. Multilevel characteristics having more than two resistance states can be an effective way to increase storage density besides device size scaling [42, 92]. Homogeneous switching dependent on device area guarantees a sufficient current to maintain reliable operation, whereas filamentary may suffer from switching instability in a scaled down device [84]. Nevertheless, filamentary exhibits superior retention due to the aligned conducting channel, since in homogenous switching, oxygen vacancies from interface tend to diffuse back to the bulk through the grain boundaries leading to poor retention performance [91].

##### Origin of Conducting Filament

CF is formed in ECM cell when a positive bias applied on an active electrode that leads to an anodic dissolution at anode/storage material interface, resulting in metal cations diffusing toward the opposite electrode [93]. The cation transfer and mobility are controlled by electron dose, Joule heating, and structural quality of the storage material [93, 94]. Mobility also controls the reduction process occurring either before or after the cations reach the opposite electrode [43, 93]. These cations then reduce in the form of tiny metal clusters that grow from anode to cathode or vice versa depending on the mobility [43, 93, 95].

Thus, the construction of these tiny metal clusters across the storage material leads to the formation of a conducting metal bridge (filament) that behaves as an electron conduction channel between the electrodes [93, 94]. During reset or rupture process, Joule heating is mostly dominated at the narrowest part of the filament, which is most known as the filament dissolution process [43, 95].

Unlike ECM cell that relies on metal cation migration from active anode, the formation of CF in VCM cell is due to the migration of oxygen ions and oxygen vacancy defects that generated within the storage material itself. When a positive bias is applied on anion-active anode, oxygen ions move toward anode; reversely, oxygen vacancies move toward cathode [43]. The percolation of oxygen vacancies across the storage material acts as an acceptor for electron carriers [43]. Yet, the physical mechanism of CF formation in VCM is a good area of research.

Kwon et al. [96] suggest that CF in TiO_2_ system is an ordered structure, Magnéli phase, that is spontaneously formed under electric field and thermal effect. Magnéli phase possess high electron conductivity near room temperature [97]. However, Kwon et al. [98] argue that the formed Magnéli phase is just as a virtual electrode while set and reset processes are the rejuvenation and dissolution of Wadsley defects, respectively. Wadsley defect can be considered as a missing plane of oxygen atoms in TiO_2_ rutile structure [98]. Similarly, Yoon et. al. [99, 100] suggest that a localized TiO_2 − x_ layer may act as an active switching region where as rejuvenation and dissolution of Magnéli phase as a switching filament may be insignificant. Nevertheless, formation of a certain conducting phase, like a Magnéli phase in TiO_2_, may not be possible in ZnO since it has no stable suboxide phase [101]. Therefore, other mechanisms on the formation of CF could be dominant.

Recent theoretical studies [102, 103] on the formation of CF in ZnO VCM cell suggest that the generated oxygen vacancies moves toward cathode and transform their 2+ charges to neutral thus weaken the Schottky barrier at ZnO/Pt interface, meanwhile the Zn^2+^ is reduced to O-deficient Zn ions (Zn^(2-n)+^) around the respective region. The chemical reaction for these processes can be expressed as [102];

1 $$ {O}_o^x\to VOoo+1/2{\mathrm{O}}_2+2{e}^{-} $$

2 $$ VOoo+2{e}^{-}\to {V}_{\mathrm{o}}^x $$

3 $$ {\mathrm{Zn}}^{2+}+{\mathrm{ne}}^{-}\to {\mathrm{Zn}}^{\left(2-n\right)+} $$

where ${O}_{\mathrm{o}}^x,\ VOoo,\ {\mathrm{O}}_2,\ {e}^{-},\ {V}_{\mathrm{o}}^x,\ {\mathrm{Zn}}^{2+},\ \mathrm{and}\ {\mathrm{Zn}}^{\left(2-n\right)+}$ are a neutral charge of oxygen ion in O site, a doubly positive charge of O vacancy, oxygen gas, a singly negative charge of an electron, a neutral charge of oxygen vacancy, a doubly positive charge of zinc ions, and a reduced positive charge of zinc ions, respectively. Equations 1, 2, and 3 are chemical reaction for the generation of oxygen vacancies, transformation of oxygen vacancies to neutral state, and reduction of zinc metal ions, respectively.

The aligned neutral oxygen vacancy (neutral oxygen vacancy filament), therefore, leads to formation of high conductive Zn^(2 − n)+^ filament [102]. Thus, the electrons prefer to conduct trough this metallic filament due to the lower chemical valence state [102, 103]. Conversely, the transformation of oxygen vacancies charge from 2+ to neutral by opposite voltage polarity (bipolar) or Joule heating (unipolar) will lead to oxygen vacancies diffuse from their configuration to other sites, in other words, disruption of filament made by oxygen vacancy [102].

**Fig. 4:**
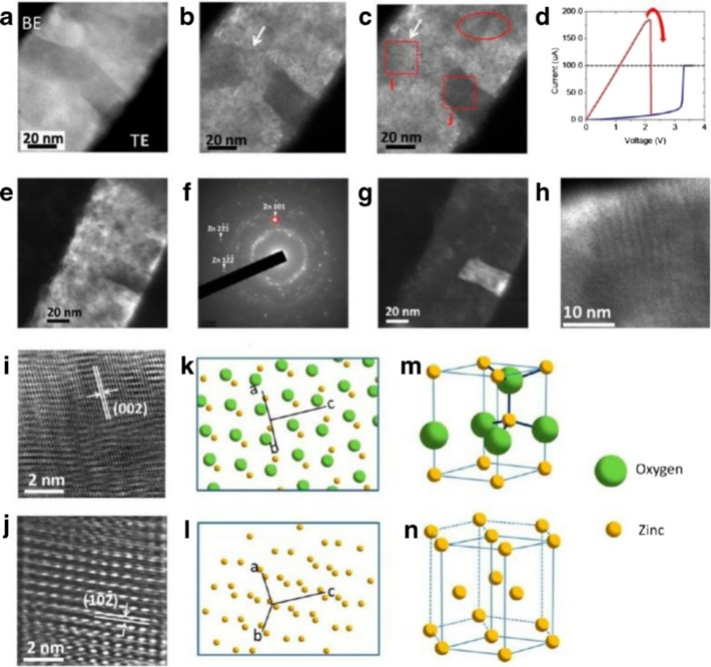
In situ TEM images of the reset process using the unipolar resistive switching method. **a** The start of recording; **b** intermediate state; **c** final state of the ruptured filament after the reset process. **d** The corresponding I-V curve in *red*; the *blue line* corresponds to the forming process as a comparison. **e** The other conductive filament in the same in situ specimen, indicating that the switching behavior is caused by multi-filament formation and rupture. **f** The selected area diffraction pattern of the conductive filament in Fig. 2e. The Zn (101) diffraction spot is marked with the *red circle*. **g** The corresponding dark-field image obtained from the diffraction spot marked as a circle in the diffraction pattern (**f**). **h** The Moire fringes can be observed at the disrupted region from a high-magnification TEM image. **i** The HRTEM image along the (110) zone axis in the disrupted region, revealing that the conductive filaments were converted back to ZnO_1 − x_. **j** The HRTEM of the “zinc” conductive filament along the (231) zone axis has been identified. **k** Solid-sphere model of ZnO in a wurtzite structure along the (110) zone axis. The coordinate lines are the unit cell vectors. **l** Solid-sphere model of zinc in a HCP structure along the (231) zone axis. The three-dimensional schematic illustrations of **m** a ZnO unit cell and **n** a zinc unit cell, respectively, showing that the zinc atoms position remain the same as the oxygen ions diffuse out [89]

Chen et al. [69, 89] directly observed CF in ZnO cell by utilizing in situ TEM (transmission electron microscope) technique. It was observed that once the oxygen vacancies reach a certain critical density, a newly generated ordered crystalline phase is formed. Figure 4 shows the reset process and structure identification of a CF in Pt/ZnO/Pt cell [89]. It was found that the CF region was identified as a Zn-dominated ZnO_1 − x_ metallic phase [89], confirming the formation of zinc metallic filament [102, 103]. This metallic phase can be ruptured when the oxygen ions migrate to this metallic phase region and convert the Zn-dominated ZnO_1 − x_ phase back to ZnO phase [69, 89]. Consequently, this evidence shows that the oxygen ion migration plays a critical role in the formation and disruption of a CF.

Despite various advance imaging techniques that have been employed to understand the nature of CF in both metal oxide-based ECM and VCM cell such as scanning transmission x-ray microscope (STXM) [104–106], TEM [69, 89, 93, 94, 96, 98, 107, 108], and C-AFM (conductive-atomic force microscope) [56, 68, 77, 78, 109–114], the relationship between retention behavior and evolution of CF is still less discussed.

#### Effect of Electrodes in ZnO-Based RRAM Devices

ZnO is generally n-type high-bandgap semiconducting materials. Although the origin of its electron conductivity is still debatable, Zni (zinc interstitial) and Vo (oxygen vacancy) defects are considered to be responsible for the low resistance [115, 116]. Abundant amount of these defects may result in insufficient switching properties. Therefore, several attempts have been reported to improve ZnO as a switching layer, such as stacked with various metal electrodes [117–128], controlled its growth deposition [52, 129–133], post-thermal treated [134, 135], doped with various elements [56, 85–87, 90, 91, 110, 136–152], and embedded/multilayered with various metalsor oxides [55, 83, 153–159].

According to electrochemical behavior of electrodes, the types of the electrodes that commonly stacked with storage material are inert, oxidizable, and active metals. Inert electrode, such as Pt, Ru, or Au, as a cathode may create high interface barrier to induce resistive switching properties [160]. High work function of these inert electrodes attributed to higher ON/OFF ratio [161]. As an anode, however, the high work function may not play important role; nonetheless, due to its inert behavior, the electrode has good electrochemical behavior that leads to efficient redox reaction [127]. Unfortunately, the preservation of oxygen in inert electrode is limited [37, 162, 163]. Unlike inert electrode, oxidizable metal electrode, as an anode, has an advantage of having oxygen reservoir behavior. This metal electrode tends to form thin interfacial metal oxide layer at top electrode (TE)/ZnO interface. The interfacial layer controls the oxygen outflow to the environment during SET process; thus the well-preserved oxygen leads to long endurance [164]. However, even though there are a number of oxidizable metal available, still, an appropriate anode for ZnO-based resistive memory needs to be selected carefully.

**Fig. 5:**
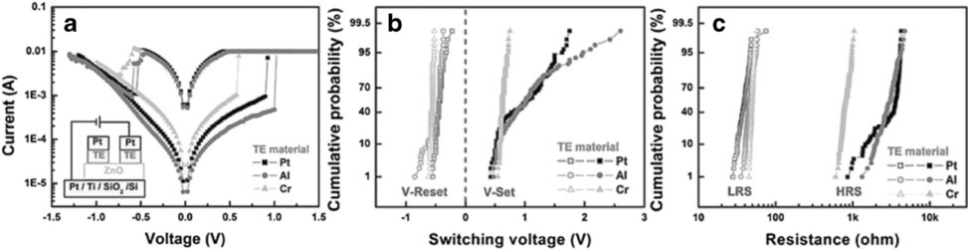
**a** Typical I-V characteristics of various electrode materials with TE/ZnO/Pt structures. The *inset* shows the schematic structure for electrical measurement of TE/ZnO/Pt structures. **b** The cumulative probability of V-Set and V-Reset. **c** The cumulative probability of HRS and LRS [163]

I-V switching characteristics of various metal top electrodes based having TE/ZnO/Pt structure is shown in Fig. 5a. Despite both Al and Cr are able to create AlO_x_ and CrO_x_as oxygen reservoir at the interface, respectively, however, obvious device instability is exhibited in devices made with Al electrode, as shown in Fig. 5b, c. Similar standard free energy of formation of oxide $\left(\varDelta {G}_f^o\right)$ between Cr and Zn leads to efficient redox process [163]. Conversely, large difference $\varDelta {G}_f^o$ between Al and Zn leads to less oxygen can be supplied from AlO_x_ to rupture the oxygen vacancies filament during reset process; in addition, the morphology of AlOx interface layer is found to be rough, which leads to device instability [163]. Hence, oxidizable metal having close $\varDelta {G}_f^o$ with ZnO and smooth interfaces are crucial in selecting appropriate anode for reliable ZnO RRAM device [163, 165]. Among these three top electrodes (Cr, Al, and Pt), Cr top electrode shows better performance compared to others.

In ECM devices, however, the resistive switching behavior depends upon the electronegativity and ionic size of the active anodes which determine the mobility of metal cations inside ZnO [161]. The use of low electronegativity and small ionic size of the active anodes result in easier formation and rupture of CF in ECM devices compared to VCM devices [161]. Therefore, set and reset voltages in ECM are lower than that in VCM. Nevertheless, it is reported that a high electronegativity of Au may also behave as active metals [108]; in addition, the omission of Ni or Ag atoms diffusion in CF formation is also reported [166, 167]; this phenomena may relate to different ZnO film quality, device geometry, and operation method. The major device parameters as a function of different metal electrodes are summarized in Table 1. The best reported structures having a good combination between low power, endurance, and retention performance so far are Pt/ZnO/Pt [122] and Ag/a-ZnO/Pt [132] for VCM and ECM devices, respectively.

**Table 1:** ZnO-based RRAM fabricated with various metal electrodes in published literature

No	Structure	CC (mA)	*V*_F_ (*V*)	*V*_R_ (*V*)	*V*_S_ (*V*)	Mode	Endurance (cycles)	ON/OFF ratio (times)	Retention (seconds)	Stress (seconds)	Memory type	Ref.
1	Pt/ZnO/Pt	30	~3.3	−1	~−2	U	100	10^3^–10^4^	NA	NA	VCM	[118]
2	Pt/ZnO/Pt	3	~4	~−0.5	~1.2	B	10^2^/10^6^(AC)	>10^2^	>6 × 10^5^/RT	NA	VCM	[122]
3	Pt/ZnO/Pt	NS	NS	−2.5	2.5	R	100	>40	NA	NA	VCM	[126]
4	Pt/ZnO/Pt	10	~4	~0.5	~1.5	U	200	58	9 × 10^4^/RT	NA	VCM	[127]
5	Pt/ZnO/Ru	10	~4	~0.7	~1.9	U	200	175	9 × 10^4^/RT	NA	VCM	[127]
6	Ru/ZnO/Pt	10	~4	~1	~2.1	U	200	61	9 × 10^4^/RT	NA	VCM	[127]
7	TiN/ZnO/Pt	5	FF	~−1.2	~1.2	B	>500	10	NA	10^5^/RT	VCM	[119]
8	Au/ZnO/ITO	SC	FF	~2	~−2	B	>10^2^	>10	10^4^/RT	NA	VCM	[120]
9	Al/ZnO/Al	1	NS	~0.5	~2.5	U	219	10^4^	10^3^	10^3^/RT	VCM	[117, 121]
10	Al/ZnO/P++−Si	5	1.56	0.27	~1.41	U	>400	>10^3^	NA	NA	VCM	[123]
11	TiN/ZnO/TiN	~80	FF	3	−4	B	NA	>10	10^4^	NA	VCM	[133]
12	Ag/ZnO/Pt	10	FF	~−0.4	0.8	B	40	10^2^	NA	10^4^/RT	ECM	[124]
13	Ag/ZnO/Cu	NS	2.5	~−1.3	~1.3	B	>500	10^3^	NA	NA	ECM	[125]
14	Cu/ZnO/ITO	NA	4.42	0.6	2.6	U	300	>20	NA	NA	ECM	[128]
15	Ag/a-ZnO/Pt	0.5	FF	−2	<0.5	B	100	10^7^	10^6^/RT	NA	ECM	[132]

Unless specified, endurance was measured using DC voltage sweeping mode

*CC* current compliance, *V*_*F*_ forming voltage, *V*_*R*_ reset voltage, *V*_*S*_ set voltage, *SC* self-compliance, *FF* forming free, *U* unipolar, *B* bipolar, *RT* measured at room temperature, *NA* data not available, *NS* not specified

#### Effect of Deposition Parameter in ZnO-Based RRAM Devices

Besides electrodes, microstructural properties and defects in ZnO film strongly affect the switching behavior as well. Zinc interstitial and oxygen vacancy native defects behave as self dopants in pure ZnO [115, 116, 168]. Excessive defect concentration leads to high leakage current and degradation of device performance [169]. Controlled ZnO film growth is required in order to fabricate good quality film having highly oriented growth and less native defects. Several methods have been reported to fabricate high-quality ZnO film for RRAM application, such as ALD [133, 170–172], MOCVD [173], PLD [135], electrospray [125], electrodeposition [167], spin-coating [174], DC-sputtering [129, 131], and RF-sputtering [52]. Yet, sputtering is the most commonly used technique due to their thickness controllability, large area uniformity, low temperature, and less-toxic process. ZnO film properties can be simply controlled by modulating Ar/O_2_ flow ratio during sputtering.

**Fig. 6:**
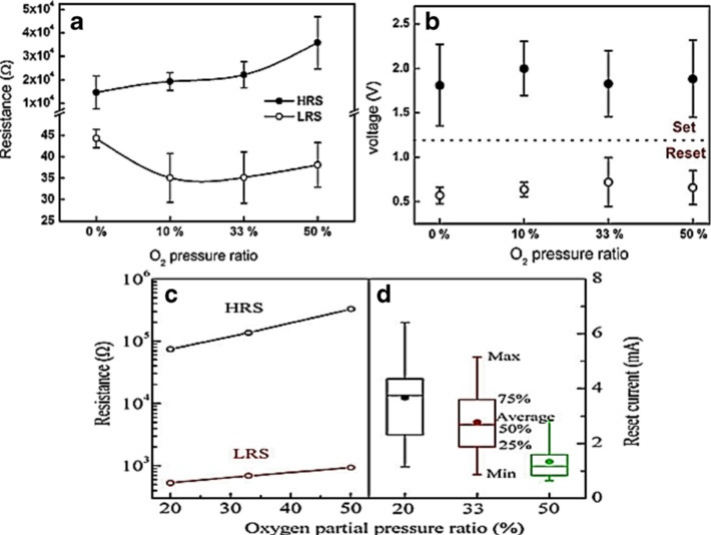
Distribution of (**a**) set/reset voltages and **b** resistance ratios of HRS/LRS at different O_2_ pressure ratio in Pt/ZnO/Pt device [52]. **c** Average HRS and LRS resistances and **d** distribution of reset current for ZnO deposited at different oxygen contents in Cu/ZnO/n^+^-Si device [129]

Figure 6 depicts the switching parameter of VCM and ECM unipolar devices made with various Ar/O_2_ flow ratio. ON/OFF ratio tends to increase as oxygen flow ratio increase. ZnO grown on higher oxygen flow condition reduces the formation of oxygen vacancy defects which can generate more free carriers, thus leading to higher HRS resistance. In terms of device stability, as oxygen flow increases VCM, devices tend to be unstable. As oxygen flow increases, smaller grains are grown in the ZnO film that leads to higher number of grain boundaries and multiple conducting path [52], leading to unstability. Excessive conductive filaments result in unstable set/reset process in RRAM devices [54]. Conversely, higher oxygen flow results in better stability in ECM devices due to lower amount of pre-existing oxygen vacancy defects in ZnO film; thus, the electron conduction controlled by metallic bridge will be more dominant than the oxygen vacancies in ECM devices. Therefore, less reset competition between the metal bridge and oxygen vacancies during Joule heating process may lead to better stability. In addition, post-thermal treatment after deposition can also be employed to improve crystallinity and adjust the defect concentration in ZnO film. The decreasing of the native defects and increasing of crystallinity in ZnO film after air or oxygen ambient annealing may enhance ON/OFF ratio in ECM and VCM devices [134, 135]. Yet, this treatment may also increase the forming voltage; a high forming voltage may generate large size and excessive number of CFs that may lead to switching instability [134, 135].

**Fig. 7:**
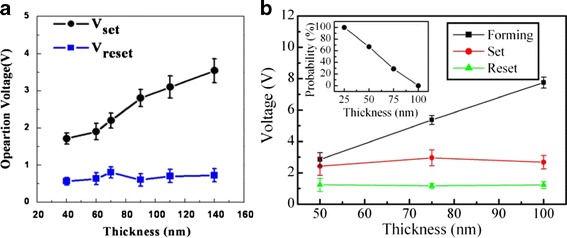
**a** Switching voltage variation of the Al/ZnO/Al structure ReRAM device a with ZnO film thickness [130], (**b**) forming, set, and reset voltages as a function of film thickness for Cu/ZnO/n^+^-Si device. Each data point was extracted from five devices. The inset of (**b**) is the thickness dependence on occurrence probability of the initially ON state for as-deposited ZnO. The occurrence probabilities were collected from 25 devices for each point [131]

Besides Ar/O_2_ flow ratio, switching layer thickness also plays a crucial role on the switching parameter in RRAM operation. Figure 7 shows the effect of ZnO thickness on the resistive switching performance of ECM and VCM unipolar devices [130, 131]. Higher forming voltage is required for thicker devices that are simply due to the longer CF that needs to be created between the electrodes. Reset voltage of VCM devices is insensitive to the thickness. However, Vset in VCM devices increases as ZnO thickness increases, due to higher crystallinity in thicker film. The higher crystallinity film having a larger grain size and lower density of dislocations may provide less conduction path during filament formation [130]. Contrarily, set and reset voltages of ECM devices are not directly affected by the structural properties due to oxide thickness. Similar phenomenon is also observed in doped ZnO bipolar ECM devices [146]. This phenomenon may arise due to the Joule heating effect taking place at a critical area where it is not significantly altered with the thickness variation [146]. It is also important to note that the improvement of ON/OFF ratio after slight adjustment in Ar/O_2_ ratio or post-annealing treatment seems more obvious in VCM than ECM devices. Consequently, it opens another area and challenge on how to modulate the Joule heating effective region in ECM cell.

#### Effect of Doping in ZnO-Based RRAM Devices

Nevertheless, controlled deposition parameter and post-thermal treatment on resistive layer may be not as effective as doping technique to fully adjust the defect concentration. Various dopant elements, such as Al [137, 175, 176], B [177], Co [138, 139, 169, 178], Cr [110, 158], Cu [87, 140, 179], Fe [180, 181], Ga [112, 182, 183], La [144], Li [184, 185], Mg [55, 111, 145, 186–190], Mn [91, 146–148, 191–195], N [56, 149], Ni [196], S [197], Sn [90, 198], Ta [199], Ti [150, 151], V [85], and Zr [86], that have been reported may exhibit decent switching performance. ZnO-based RRAM with multi-element doping, such as Al-Sn [136, 200], Ga-Sn [201], and In-Ga [141–143, 202–208], is also proposed. The effect of doping on the resistive switching performance is summarized in Table 2. Among the reported devices, devices having Mn dopant exhibit not only good endurance but also long retention performance.

**Table 2:** Doped ZnO-based RRAM in published literature

No	Structure	CC (mA)	*V*_F_ (*V*)	*V*_R_ (*V*)	*V*_S_ (*V*)	Mode	Endurance (cycles)	ON/OFF ratio (times)	Retention (seconds)	Stress (seconds)	Ref.
1	Cu/N:ZnO/Pt	10	NS	~−0.45	~1.47	B	100	>10^2^	NA	NA	[56]
2	Ag/Zn_0.98_Cu_0.02_O/ITO	10	NS	~−0.02	1.8	B	NA	10^6^	<10^3^	NA	[87]
		1	NS	−3.5	−15	U	NA	10^4^	>10^3^	NA	
3	Pt/ZnVO/Pt	10	~−4	~−0.5	~−2.5	B	10^5^	~10^2^	36 × 10^3^/85 °C	NA	[85]
4	Pt/Zn_0.99_Zr_0.01_O/Pt	1	~−2	~1	~−1.5	B	10^4^	~10^2^	NA	NA	[86]
5	Al/ZTO/Pt	5 × 10^−4^	~−2	~1	~−2.5	B	50	1.4 × 10^3^	10^4^/RT	NA	[90]
6	Pt/Mn:ZnO/Si	NS	FF	~−20	~20	B	45 × 10^2^	~ 10^3^	5 × 10^3^/RT	NA	[91]
7	Pt/Zn_1 − x_Cr_x_O/Pt	–	FF	~3.5	~3	B	100	7 × 10^3^	36 × 10^3^/RT	NA	[110]
8	Ti/AZTO/Pt	3	NS	~1.6	~1.1	B	256	18	NA	10^4^	[136]
9	Au/Co-ZnO/ITO/Au	–	FF	4	2.6	BS	4000	~7	NA	NA	[138]
10	Pt/Co:ZnO/Pt	10	FF	~−1	1.5–3	B	300	10^2^	NA	NA	[139]
11	Al/ZnO:Cu/Pt	10	~12	~0.5	~2	U	450	470	10^4^/RT	NA	[140]
12	TiN/Ti/IGZO/Pt	10	~6	~−1.5	~1	B	10^4^	~10	NA	NA	[141]
13	Pt/a-IGZO/Pt	10	~10	~−1	~1.5	B	100	>10	>10^4^/RT	NA	[142]
14	Al/IGZO/Al	–	~5	~5	~−5	B	100	~2	NA	NA	[143]
15	Pt/ZnLaO/p-Si	10	~6	~1	~2.5	U	150	>10	10^6^	NA	[144]
	Pt/ZnLaO/Pt	10	~4	~0.5	~2.5			>10^3^		NA	
16	Pt/(Zn_1 − x_Mg_x_)O/Pt	10	~5	~1.5	~3.5	U	50	140–10^3^	NA	NA	[145]
17	Cu/ZnO:Mn/Pt	5	~1.9	~−0.6	~1.2	B	65	>10^3^	10^4^/85 °C	NA	[146]
18	Au/ZnMn_2_O_4_/Pt	1	NS	~2	~10	U	8000	10^5^–10^7^	4 × 10^4^/RT	NA	[147]
	Au/ZnMnO_3_/Pt							10^4^–10^5^			
19	Pt/Mn:ZnO_x_S_1 − x_/Cu	NS	NS	~0.5	1–3	U	100	10^5^–10^6^	10^4^/RT	NA	[148]
20	Au/Cr/ZnO:N/TiN	5	2.5	~−1	~0.75	B	100	~1	10^4^/RT	NA	[149]
21	Au/Ti:ZnO/ITO	–	FF	~−3	~3	B	200	14	2 × 10^3^/RT	NA	[150]
22	Pt/ZnO:Ti/n+–Si	10	~5.5	~1	2–4	U	200	>10^2^	>10^5^/RT	NA	[151]
23	Ag/ZnO:Mn/Pt	SC	FF	−2.6–−0.5	0.3–3.8	B	100	10^7^	>10^7^/RT	NA	[152]
24	Al/GaZnOx/p^+^-Si	7	~4.8	~−2.8	~3.5	B	100	10^2^	NA	NA	[183]
25	Ti/Mg_0.1_ZnO_0.9_/Pt	1	FF	−1.5	1.5	B	500	>10^3^	10^4^	NA	[189]

*NS* not specified, *CC* current compliance, *SC* self-compliance, *FF* forming free, *U* unipolar, *B* bipolar, *BS* bistable, *VF* forming voltage, *VR* reset voltage, *VS* set voltage, *RT* measured at room temperature, *NA* data not available

The concentration of native and extrinsic defects induced by doping can be efficiently tuned by considering the defect generation chemistry. The formation of native defects in nonstoichimetric ZnO can be expressed using Kroger-Vink notation as follows [169, 209]:

4 $$ {\mathrm{Zn}}_{\mathrm{Zn}}^x+2{O}_o^x\leftrightarrow \mathrm{Z}\mathrm{n}ioo+{\mathrm{O}}_2(g)+2{e}^{\prime } $$

5 $$ {O}_o^x\leftrightarrow \frac{1}{2}{\mathrm{O}}_2(g)+VOoo+2{e}^{\prime } $$

where ${\mathrm{Zn}}_{\mathrm{Zn}}^x$ and $\mathrm{Z}\mathrm{n}ioo$ are a neutral charge of a Zn ion in a zinc site and a doubly positive charge of a Zn ion in an interstitial site, respectively. Excessive $\mathrm{Z}\mathrm{n}ioo$ and $VOoo$ concentration may deteriorate switching performance [145, 169]. Therefore, the purpose of ZnO doping is to promote compensator defects and to decrease the native defect concentration. For example, the formation of compensator defects due to Co dopant can be expressed using Kroger-Vink notation as follows [169, 209]:

6 $$ {\mathrm{Co}}_{\varkappa }{O}_{\gamma}\underleftrightarrow{\mathrm{XZnO}}\varkappa {\mathrm{Co}}_{Zn}^x+\varkappa {O}_o^x+\left(\gamma -\varkappa \right)\ \left(Oi\hbox{'}\hbox{'}+2{h}^o\right) $$

7 $$ {\mathrm{Co}}_{\varkappa }{O}_{\gamma}\underleftrightarrow{\mathrm{YZnO}}\varkappa {\mathrm{Co}}_{\mathrm{Zn}}^x+\gamma {O}_o^x+\left(\gamma -\varkappa \right)\ \left({V}_{\mathrm{Zn}}^{\hbox{'}\hbox{'}}+2{h}^o\right) $$

where ${\mathrm{Co}}_{\mathrm{Zn}}^x,{O_i}^{\prime \prime },{V_{\mathrm{Zn}}}^{\prime \prime }$, and *h*^*o*^ are a neutral charge of Co ion in a Zn site, a doubly negative charge of an O ion in an interstitial site, a doubly negative charge of a Zn vacancy, and a singly positive charge of a hole, respectively. As the result, oxygen concentration and insulating behavior in resistive layer are increased and thus improve ON/OFF ratio [169]. However, excessive dopant may deteriorate switching cycles and stability performance [110, 143, 151, 169, 175, 179, 190]. The deterioration occurs due to the weakening of c-axis-textured structure after increasing dopant concentration [169].

**Fig. 8:**
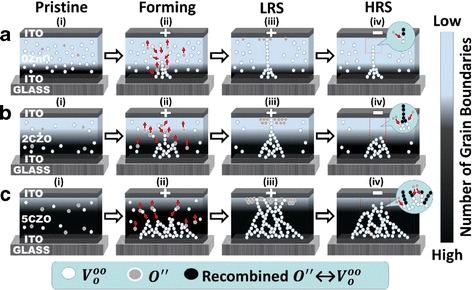
Schematic of switching mechanism of Zn_(1-x)_Co_x_O RRAM with (**a**) 0 mol%, (**b**) 2 mol%, and (**c**) 5 mol% of CoO dopant concentration [169]

C-axis texture is a beneficial characteristic of ZnO that plays an important role to confine CF [127]. Figure 8 shows the schematic of CF development in various concentration of Co-doped ZnO resistive layer [169]. The study suggests there is a trade-off in reducing native defects and maintaining c-axis-textured structure in ZnO-based RRAM [169]. Therefore, the trade-off should be well adjusted for achieving a decent endurance performance.

It can also be noted that such trade-off may limit the fabrication of fully c-axis-textured growth. Consequently, the “hunt” for finding suitable doping element and technique that allow the increasing of both acceptor defects and microstructural quality is still needed. Employing a p-type doping element such as nitrogen may increase both acceptor concentration and microstructural quality, thus improving RRAM performance [56, 149]. In addition, p-type ZnO-based RRAM still has not received sufficient attention yet.

Employing an amorphous resistive layer may also avoid the formation of excessive and branching of CF due to the lack of grain boundary structure. Several efforts have been reported to fabricate amorphous ZnO-based RRAM, such as by doping [90, 141–143, 177, 198, 201–203, 205–208], hydrogen peroxide treatment [183], and deposition parameter optimization [132].

#### ZnO-Based Complementary RRAM

Although doping technique can be considered as the simplest way to improve switching properties, embedded and multilayered structure still receive great attention due to easy modulation of switching behavior in the switching layer. Table 3 shows the summary of the important switching parameters of multilayered and embedded ZnO-based RRAM. Multilayer structure can be employed not only to improve switching performance but also to generate different peculiar switching characteristics. By employing proper electrical programming to control formation and rupture of CF at the particular switching layer, complementary switching (CS) characteristics can be achieved. CS is a unique switching characteristic that is useful for avoiding sneak path disadvantage in a three-dimensional crossbar RRAM application [153, 154].

**Table 3:** Multilayered and embedded ZnO-based RRAM in published literature

No	Structure	CC (mA)	*V*_F_ (*V*)	*V*_R_ (*V*)	*V*_S_ (*V*)	Mode	Endurance (cycles)	ON/OFF ratio (times)	Retention (seconds)	Stress (seconds)	Ref.
1	Pt/ZnO/Ag_0.2_-Al_0.8_/Al	1	NS	~0.3	~2	U	200	>10^2^	NA	NA	[53]
2	TiN/MgZnO/ZnO/Pt	10/20	~6	−2	~1	B	10^4^	>50	3 × 10^4^/RT	NA	[55]
3	Pt/ZnO/ZrO_2_/Pt	10	~−6.5	−4	~3	B	100	~5	NA	10^4^	[83]
4	Pt/ZnO/CoOx/ZnO/Pt	10	FF	0.8–1.8	1.5–2.9	B	200	~10^2^	NA	NA	[155]
5	Pt/ZnLaO/ZnO/Pt	10	~3.5	~1	~2.3	U	100	~10^4^	10^4^/65 °C	NA	[156]
6	Ag/CeO_2_/ZnO/NSTO	10	NS	−5	~2	B	100	540	10^3^/RT	NA	[157]
7	Pt/ZnO/Cr/ZnO/Pt	NS	~2	−2	3	B	10^4^	~10^4^	NA	5 × 10^3^	[158]
8	Pt/(ZnO/Ti/ZnO)_1–4_/ITO	NS	FF	~−2.5	~2	B	320	~10^3^	>10^6^	NA	[159]
9	Ag/GZO/ZnO/Pt/Ti	10	FF	0.55	0.4	B	~40	2 × 10^3^	1.1 × 10^4^	NA	[182]
10	Pt/TiO_x_/ZnO/n^+^-Si	NS	~2.8	~0.5	~2	U	>50	>10^2^	NA	NA	[286]
11	Al/Al_2_O_3_/(ZnO/Al_2_O_3_)10/n-Si/Al	NS	FF	−7	7	B	NA	10^3^–10^4^	10^3^	NA	[287]

*NS* not specified, *CC* current compliance, *FF* forming free, *U* unipolar, *B* bipolar, *VF* forming voltage, *VR* reset voltage, *VS* set voltage, *RT* measured at room temperature, *NA* data not available

**Fig. 9:**
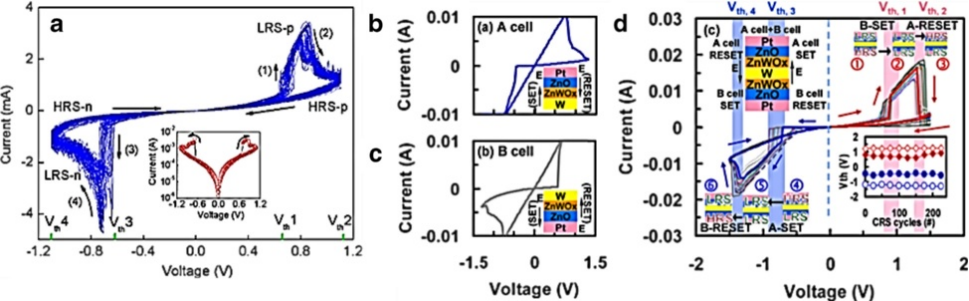
**a** Typical complementary switching of TiN/MgZnO/ZnO/Pt bipolar memory device. *Inset* shows I-V curve of one typical switching process plotted in semilogarithmic scale [153]. **b** I-V behaviors of cell A (Pt/ZnO/ZnWOx/W) and **c** cell B (W/ZnWOx/ZnO/Pt). *Inset* shows the corresponding device configuration. **d** I-V sweeps of the ZnO/ZnWOx//ZnWOx/ZnOCRS device. *Inset* shows the device configuration and the corresponding resistive switching for two cells. All the thickness of ZnWOx layer is ∼15 nm [154]

Figure 9a shows the CS characteristic of TiN/MgZnO/ZnO/Pt double layer memory device [153]. The CS characteristic can be obtained under proper programming steps, which can vary from device to device. For the TiN/MgZnO/ZnO/Pt memory device structure, the programming steps are as follows [153]: firstly, the device is operated under common counterclockwise bipolar switching mode. When the device is on LRS (set), low negative bias of −1 V was applied on top electrode (TE); consequently, oxygen vacancies move toward the MgZnO layer, by leaving some filament gap in the ZnO layer and make the device in HRS (reset). This process is called second electroforming. When low positive bias of ~0.6 V is applied on TE (V_th1_), the oxygen vacancies repulse back to ZnO layer and resulted both layers in LRS (set); however, as the bias is continuously applied to reach ~1 V (V_th2_), the oxygen vacancy filament size at MgZnO layer is reduced and ruptured. Similarly, the same mechanism may apply with the negative bias. Low negative bias of ~−0.6 V (V_th3_) may repulse the oxygen vacancies from ZnO layer to MgZnO layer and form the filament at the MgZnO layer; consequently, both layers are in LRS (set). As the negative bias is continuously applied to reach ~−1 V (V_th4_), the oxygen filament size at ZnO layer is gradually ruptured and makes the device back to HRS (reset) [153].

Another method to produce CS characteristic is by simply reversely stacking two cell memories. Figure 9b, c shows the resistive switching characteristics of Pt/ZnO/ZnWOx/W (cell A) and W/ZnWOx/ZnO/Pt (cell B) memory, respectively [154]. These devices can only be set (LRS) and reset (HRS) by applying negative and positive bias at the Pt electrode, respectively. Figure 9d shows the CS characteristics of Pt/ZnO/ZnWOx/W/ZnWOx/ZnO/Pt device [154]. The CS characteristic on this device can be generated under programming steps as followed [154]; initially, both cells are in HRS, called as “OFF” state. Then, one of the cells (cell A) is switched to LRS while cell B can be maintained in HRS by sweeping low negative bias at the TE. At this stage, cell B is still in HRS because it requires the opposite bias to switch and act as a voltage divider. As the positive sweep bias reach ~0.9 V (V_th1_), cell B is switched to LRS. Because the positive bias of ~0.9 V is not enough to switch the cell A from LRS to HRS, therefore at this stage, both cells are in LRS. When both cells are in LRS, it is called as “ON” state. Further increase of positive bias to ~1.3 V (V_th2_) leads to reset cell A. At this stage, cells A and B are in HRS and LRS, respectively, called as “1” state. In order to switch the device back to “ON” state, cell A needs to switch back to LRS by sweeping with negative bias of ~−0.6 V (V_th3_); therefore, both cells are in LRS. Similarly, at this stage, cell B can still maintain its LRS because ~−0.6 V is not enough to switch it to HRS. However, as the sweep negative bias continues to reach ~−1.3 V (V_th4_), cell B is switched to HRS. Therefore, at this stage, cells A and B are in LRS and HRS, respectively, called as “0” state. Consequently, four distinct threshold biases can be applied to obtain CR switching characteristics [154].

Achieving CS by employing the above methods is very useful to maintain simplicity of the memory structure and fabrication. Yet, further investigation is necessary to expand the potential of these methods for ZnO-based ECM cell and transparent VCM cell.

#### ZnO-Based Transparent/Flexible RRAM

Nonvolatile memory structure having high transmittance in visible light would be useful for the realization of fully integrated transparent electronics [210, 211]. Employing wide direct band gap switching materials, transparent conducting oxide (TCO) electrodes, and transparent substrate is required to construct invisible RRAM structure. ITO [54, 176, 212–220], FTO [213], AZO [54, 219, 221], and GZO [222–224] are the commonly used TCO as electrodes for transparent electronics. Conduction and interfacial growth properties of TCO are strongly determined by its fabrication parameter. However, still few investigations have been conducted to explain phenomena in RRAM operation due to different properties of TCO [219, 225–227].

**Fig. 10:**
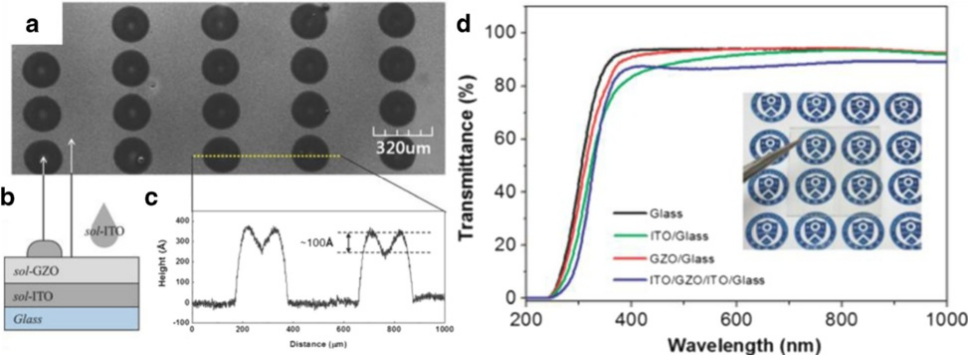
**a** UV-laser confocal image of inkjet-printed ITO electrodes on a GZO/spin-coated ITO/glass substrate. **b** Schematic of an all solution processed ITO/GZO/ITO RRAM device. **c** Surface profile of the top electrodes following the *yellow line* in (**a**). **d** UV-vis transmittance of bare glass (*black*), ITO/glass (*green*), GZO/glass (*red*), and ITO/GZO/ITO/glass (*blue*). To determine the optical properties, both the ITO bottom and top layers were spin-coated four times, resulting in a film with a thickness of ∼30 nm. The *inset* is a photograph of the sol-TRRAM (ITO/GZO/ITO/glass) device. The background is observed through the device without refraction or distortion [214]

High quality of ZnO resistive switching layer was deposited on TCO electrodes using various methods, such as metal organic chemical vapor deposition (MOCVD) [212, 223, 224], pulsed laser deposition [213, 218, 221, 222], RF-sputtering [54, 176, 216, 217, 219], hydrothermal growth [216], and sol-gel [214, 215, 220]. Figure 10a–c demonstrates sol-gel-derived ITO/GZO/ITO RRAM devices on glass substrate. The device is fully transparent (~80 %) in the visible region as shown in Fig. 10d. Devices with an average transparency of above 70 % in visible light region can be considered as having transparent structure, while semi-transparent is below 70 % [228]. Table 4 summarizes the switching parameters and performances of the ZnO-based transparent RRAM (TRRAM). Multilayer devices made of GZO/Ga_2_O_3_/ZnO/Ga_2_O_3_/GZO structures exhibit the highest transparency with high memory window and long retention performances [223].

**Table 4:** ZnO-based transparent RRAM in published literature

No	Structure	%T	Mode	CC (mA)	*V*_F_ (*V*)	*V*_R_ (*V*)	*V*_S_ (*V*)	Endurance (cycles)	ON/OFF ratio (times)	Retention (s/°C)	Ref.
1	AZO/ZnO_1 − x_/ITO	~85	B	1	−5.5/4 (DF)	−2	~1.7	>450	~10^2^	10^4^/RT	[54]
2	ITO/Zn_0.98_Co_0.02_O/ITO	90	B	5	3	−1.5	1.2	5000	15	NA	[169]
3	ITO/AZO/ITO	~81	B	10	~2.3	~−0.5	~0.5	300	3	NA	[176]
4	ITO/ZnO/ITO	81	U	15	3.2	1.8	2.6	10^2^	~10^2^	10^5^/RT	[212]
5	ITO/ZnO:Mg/FTO	80	B	50	2.8	−3	1.8	10^5^	2.5	5 × 10^3^/110 °C	[213]
6	ITO/GZO/ITO	~86.5	B	0.1	FF	−7	6	350	15	NA	[214]
7	ITO/GZO-nanorods/ZnO/ITO	~80	B	10	~3	~−2	~2	>7000	>200	10^4^/85 °C	[216]
8	ITO/graphene/ZnO/ITO	75.6	B	5	4	~−2.5	~1	100	20	10^4^/RT	[217]
9	ITO/ZnO/Pr_0.7_Ca_0.3_MnO_3_/ITO	79.6	B	10	FF	~2	~−2	2.5 × 10^3^	10^4^	NA	[218]
10	AZO/ZnO/ITO	~80	B	10	~3.5	−2	~1.5	10^4^	14	NA	[219]
12	AZO/MZO/AZO	64–82	B	1	−6	~−4	~3	50	3	10^5^/RT	[221]
13	GZO/ZnO/GZO	~80	U	10	3.5	~1.6	~2.2	7	~5	NA	[222]
14	GZO/Ga_2_O_3_/ZnO/Ga_2_O_3_/GZO	92	B	20	FF	−12	14	50	10^2^	10^5^/RT	[223]
15	ITO/IGZO/ITO	70–80	B	10	FF	3.5	~−1	10^2^	32	10^4^/RT	[288]

*%T* percentage of transmittance in visible range, *U* unipolar, *B* bipolar, *CC* current compliance, *FF* free forming, *V*_*F*_ forming voltage, *V*_*R*_ reset voltage, *V*_*S*_ set voltage, *DF* double forming, *RT* measured at room temperature, *NA* data not available

**Fig. 11:**
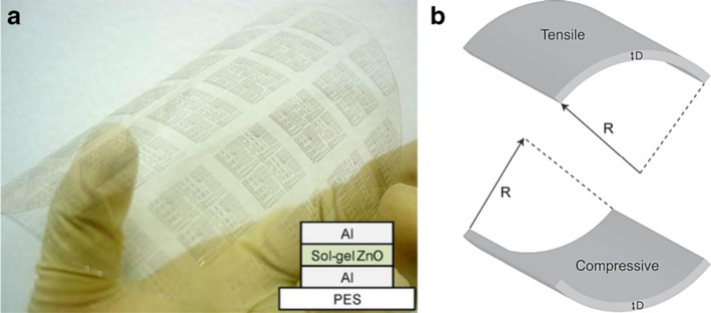
**a** Photograph of Al/ZnO/Al flexible resistive memory devices fabricated on PES substrate [232]. **b** Schematic illustrations of the flexible substrate at tensile strain (*up*) and compressive strain (*down*), where *R* is the bending radius and *D* is the thickness of the substrate [238]

By taking an advantage of low synthetic temperature of ZnO material, it allows us to fabricate flexible RRAM (FRRAM) devices on polymer substrate. Flexible nonvolatile memory may revolutionize electronics due to its potential in embedded flexible technologies [229]. Polyethylene terephthalate (PET) [220, 230, 231], polyethylene naphthalate (PEN) [113], polyethersulfone (PES) [232, 233], polymide (PI) [234], and Kapton [235] are commonly used as polymer substrate; however, flexible RRAM, but not transparent, having metal foil [236] and stainless steel [237] substrates, was also reported. Figure 11a shows the typical photograph of flexible RRAM having Al/ZnO/Al structures fabricated on PES substrate.

Mechanical flexibility is the essential consideration for flexible memory application feasibility. Reliable memory window and endurance should be maintained in various bending condition and after repetitive flexes. Mechanical parameter related to tensile and compressive strain that were given upon a memory structure should be taken into account. Figure 11b shows the schematic illustration of a flexible substrate receiving tensile and compressive strain. The strain induced on the surface of a substrate due to bending can be calculated using Eq. 8 [238];

8 $$ S=\frac{\left({t}_{\mathrm{L}}+{t}_{\mathrm{S}}\right)\left(1+2\eta +x{\eta}^2\right)}{2R\left(1+\eta \right)\left(1+x\eta \right)} $$

where *S* is strain, *η* = *t*_L_/*t*_S_, *t*_L_ is layer thickness, *t*_S_ is substrate thickness, *χ* = *Y*_L_/*Y*_S_, *Y*_L_ is the Young’s modulus of the layer, and *Y*_S_ is the Young’s modulus of the substrate. Nevertheless, there is no definite standard or apparatus for mechanical flexibility test on RRAM; yet, HRS and LRS values recorded at different bending radius and repeated bending are generally conducted to measure the memory flexibility.

Employing metal as bottom electrode may help mechanical flexibility of FRRAM due to its higher ductility properties as compared to oxide electrode. Nonetheless, having TCO as electrodes is unneglectable when fabricating transparent and flexible RRAM (TFRRAM). TCO films fabricated on flexible substrate may suffer from cracking after repeated bending. Related to that, compressive stress results more damage to the films than tensile stress [239]. In order to minimize this issue, inserting thin metal between TCO layers has been suggested [233].

**Fig. 12:**
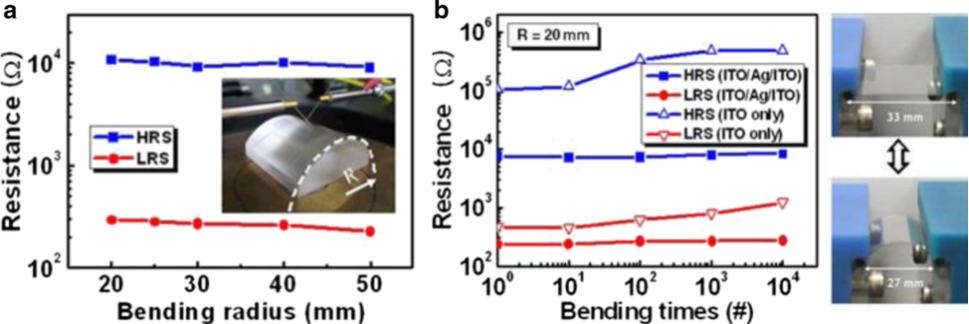
**a** Bending effect of the TFRRAM device. Photograph (shown in the inset) of the device bent at *R* = 30 mm. **b** Continuous bending effect of the TRRAM device [233]

Figure 12 shows flexibility test of ITO/ZnO/ITO/PES and ITO/ZnO/ITO/Ag/ITO/PES TFRRAM. Both devices have shown stable states in various bending radius, as shown in Fig. 12a. However, devices having single layer ITO bottom electrode suffer HRS and LRS degradation upon repeated bending; conversely, devices having ITO/Ag/ITO multilayer bottom electrode exhibit excellent stability. However, inserting thin metal film between TCO layers may decrease its transparency [233] and increase the cost of production process [239]. Another method to enhance bending durability of films while maintaining the transparency is by employing TCO/oxide buffer layer/flexible substrate structure [239].

Nonetheless, mechanical flexibility of FRRAM can rely on not only the ductility of the bottom electrode but also the resistive layer as well. Interestingly, Al/GOZNs/ITO/PET FRRAM devices are able to maintain its states after repeated bending with extreme bending radius of 6 mm [230]. This may suggest that the excellent mechanical flexibility of the FRRAM may also be attributed from the ZnO nanorods-graphene oxide composite resistive layer ductility. Recent report also implied that employing amorphous InGaZnO (α-IGZO) as a resistive layer may exhibit better mechanical flexibility than polycrystalline oxide resistive layer [240]. Yet, further investigation is necessary to explain the influence of ZnO microstructural properties on resistive switching performance under various bending condition. The switching parameters of the flexible resistive switching memory along with flexibility are summarized in Table 5.

**Table 5:** ZnO-based flexible RRAM in published literature

No	Structure	%T	Mode	CC (mA)	*V*_F_ (*V*)	*V*_R_ (*V*)	*V*_S_ (*V*)	Endurance (cycles)	ON/OFF ratio (times)	Retention (s/°C)	Stress (s)	Flexibility test			Ref.
												Bending cycles (times)	Radii (mm)	ON/OFF ratio (times)	
1	GZO/GZO(H)/GZO/PEN	66	B	0.1	~1.7	−2	~1.5	20	20	NA	NA	NA	NA	NA	[113]
2	Ag/ZnO/ITO/PET	NT	B	SC	NS	3 – 4.9	−0.7–−3.2	>100	>60	>4 × 10^3^/RT	NA	2400	8	~10	[220]
3	Al/GOZNs/ITOPET	NT	B	2	NS	−2	2.1	200	~10^2^	10^4^/RT	NA	10^3^	6	~10^2^	[230]
4	Cu/ZnO:Mg/ITO/PET	NT	B	1	2.6	~−1.5	~ 1	100	30	144 × 10^2^/RT	NA	10^3^	20	30	[231]
5	Al/ZnO/Al/plastic	NT	U	5	FF	~0.5	~2	10^4^	10^4^	NA	10^5^	10^5^	NS	~10	[232]
6	ITO/ZnO/ITO/Ag/ITO/PES	80	U	10	3.4	0.6	1.5	200	>10	10^5^/85 °C	NA	10^4^	20	>10	[233]
7	Au/ZnO NR/Au/PI	NT	U	50	~1.7	0.23 ± 0.02	0.84 ± 0.04	>100	10	10^4^/RT	NA	100	20	~10	[234]
8	Al/Mn:ZnO/HfO_2_/Ti/Pt/Kapton	NT	B	NS	NS	−5	5	50	70	NA	500	500	11	~70	[235]
9	Au/ZnO/Stainless steel	NT	NP	30	NS	±0.5–0.8	±1.0–2.0	100	10^2^	NA	NA	NA	NA	NA	[237]
10	Cu/α-IGZO/Cu/plastic	~65	U	3	FF	~0.5	~1.5	150	10^2^–10^3^	NS	NA	10^5^	NS	10^2^	[240]

*%T* percentage of approximate transmittance in visible range, *NT* not transparent, *NP* nonpolar, *CC* current compliance, *U* unipolar, *B* bipolar, *SC* self-compliance, *FF* free forming, *Vf* forming voltage, *Vr* reset voltage, *Vs* set voltage, *RT* room temperature, *NS* not specified, *NA* not available

#### Sneak Current Prevention in ZnO-Based RRAM

To resolve the physical scaling issues of conventional nonvolatile memory devices, crossbar array architecture has been considered as an attractive construction due to the scalability, simplicity, and multiple stackability of the structure. For practical applications, the foremost bottleneck of this array architecture is the sneak current path issue, which leads to read operation error [241]. ZnO-based RRAM having crossbar structures has also suffered from sneak path issue. To suppress the undesired sneak current, the combination of memory cells with rectifying or switch devices, such as p-n junction diodes [242], Schottky diodes [243, 244], threshold switching devices [245], and transistors [246], is necessary. In this section, we will discuss about the different aspects to eliminate sneak path for the ZnO-based RRAM.

**Fig. 13:**
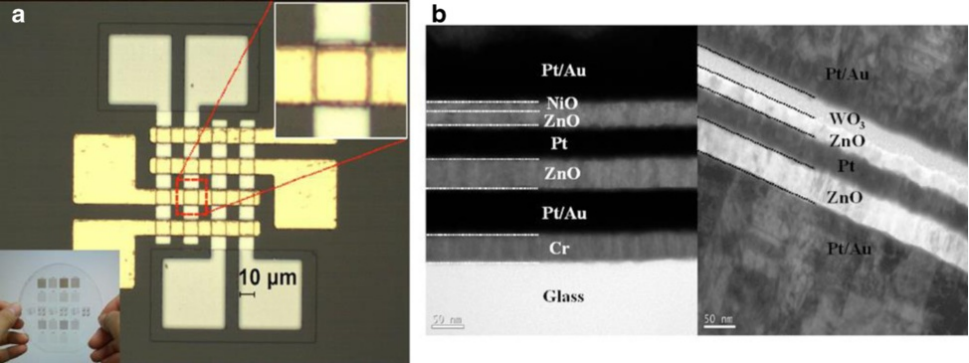
**a** A 4X4 crossbar array ZnO RRAM device stacked with heterostructure diodes. The *lower inset* is an image of the entire structure of the device. **b** Cross-sectional TEM images of the stacked contact area [247]

**Fig. 14:**
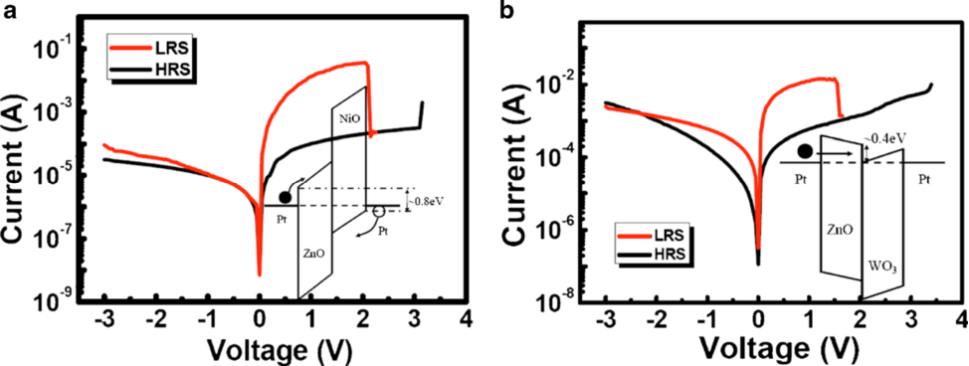
**a** I-V characteristics of the crossbar ZnO RRAM device stacked with (**a**) a ZnO/NiO p-n junction and **b** WO_3_/ZnO tunnel barrier diodes. *Inset* of **a** and **b**: equilibrium band alignment of Pt/NiO/ZnO/Pt and Pt/ZnO/WO_3_/Pt. *Solid circle* denotes electron, *open circle* denotes hole, and *arrows* denote possible conduction mechanisms of carriers. The indicated energy values in the inset diagrams are calculated based on literature values (ref. [284, 285]) [247]

**Fig. 15:**
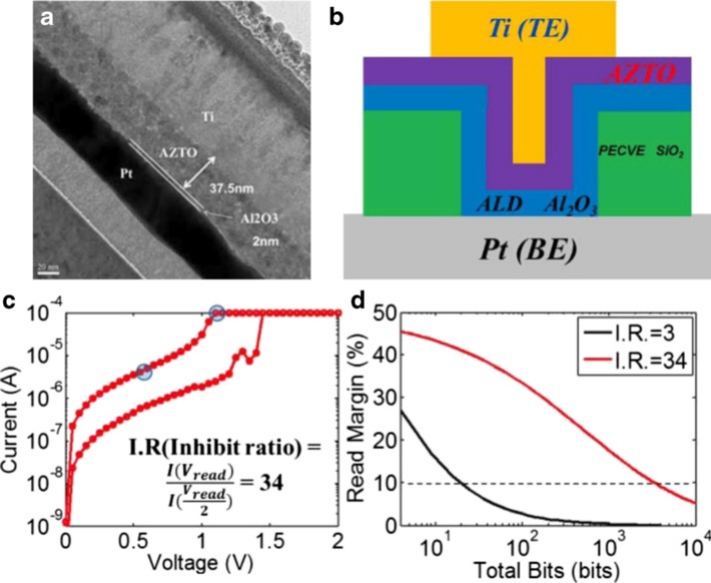
**a** Cross-section TEM image of Ti/AZTO/Al_2_O_3_/Pt structure. **b** Schematic of the fabricated memory device structure. **c** Inhibit ratio (IR) of the nonlinear AZTO-based RRAM device. **d** The read margin analysis indicate that the proposed IR can extend array to ~3K bits [200]

Seo et al. [247] reported ZnO crossbar Pt/ZnO/Pt resistive random access memory stacked with heterostructure diodes of Pt/NiO/ZnO/Pt p-n junction and the Pt/WO_3_/ZnO/Pt tunnel barrier diodes for eliminating the sneak current effect to avoid sneak path current, as shown in Fig. 13. The fabricated ZnO RRAM device on glass with a 4 × 4 crossbar array stacked with heterostructure diodes is shown in Fig. 13a. Cross-sectional TEM images of the stacked contact area of different diodes is presented in Fig. 13b. Figure 14a, b reveals the current characteristics of the crossbar array ZnO RRAM devices combined with the heterostructure diodes. Inset shows the corresponding energy band diagram of the diodes. Stable resistive switching occurring with larger operation voltages is observed due to the additional series resistance of the heterostructure diodes. However, the reverse current was effectively suppressed by combining with the diodes. Similar behavior is also reported by employing vertically integrated Ag/MgZnO/GaZnO/Au Schottky diode on Au/FeZnO/MgO/Pt RRAM device [248]. Usually, diode or transistor or selector needs to be combined with the RRAM resistor to avoid sneak path current. But, recently, Fan et al. avoided sneak path current by fabricating selector-less AZTO-based RRAM with a thin insertion layer (2 nm) of Al_2_O_3_ [200]. Figure 15a, b shows typical cross-section TEM image of Ti/AZTO/Al_2_O_3_/Pt and the schematic of the fabricated device, respectively [200]. This device is able to exhibit read margin with inhabit ratio (IR) of 34 times, as depicted in Fig. 15c, d [200]. They suggested that Al_2_O_3_ may act as an electron barrier where the LRS conduction is dominated by electron tunnelling mechanism [200].

#### Nanostructured ZnO-Based RRAM

To reduce the production cost, highly densed RRAM can be achieved with a maximum size of 4F^2^ high packing density by stacking architecture via three-dimensional crossbar [43, 154, 249]. However, further effort to scaling down the memory size using unique structure, such as self-assembly nanostructure, is very attractive. The wide variety of ZnO morphologies offers novel approach and understanding to dimensionality dependence on switching characteristics. Currently, devices employing nanorods/nanowires [58–75, 216, 250–266], nanobelts [76], and nanoisland [77, 78] as the switching elements receive considerable interests in developing one-dimensional resistive memory for ultrahigh density memories.

**Fig. 16:**
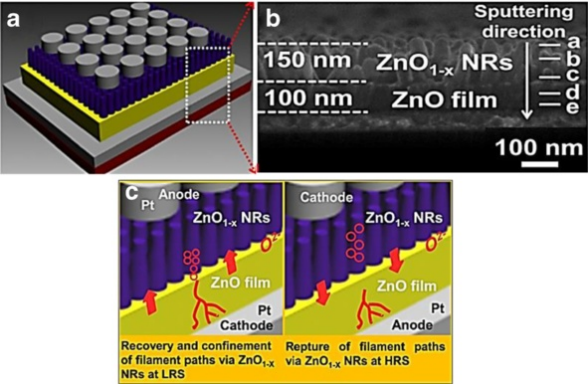
**a** Schematic of a Pt/ZnO_1 − x_ NRs/ZnO TF/Pt resistive switching device. **b** Corresponding SEM image of a well-aligned ZnO_1 − X_ NR with a length of ∼150 nm grown on the ZnO film with a thickness of ∼100 nm. **c** Schematic of confined recovery and rupture of conducting filaments by ZnO_1 − x_ NRs [256]

**Fig. 17:**
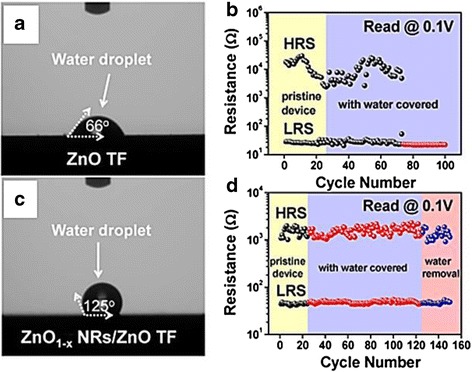
**a**, **b** Contact angle measurements for surfaces of the ZnO film and the ZnO_1 − x_ NRs, respectively. **c**, **d** Corresponding endurance tests for two devices measured with coverage of a water droplet at a read bias of 0.1 V, respectively [256]

Figure 16a, b shows the schematic of Pt/ZnO_1 − x_ nanorods/ZnO/Pt device and the cross-sectional SEM image, respectively [256]. It is believed that ionic defects prefer to diffuse through the nanorods sidewall due to higher microstructural defects [252, 253]. Thus, the vertically aligned nanorods may induce filament confinement, as depicted in Fig. 16c [256]. However, RRAM device made with ZnO nanorods layer with low packing density may suffer from short circuit problem due to direct contact between top and bottom electrodes [254]. Nevertheless, such issue can be avoided by embedding the nanorods layer in insulating polymers [253] or synthesizing highly densed nanorod film [216, 254]. In addition to that, employing nanorod layer may demonstrate surface self-cleaning function against water contact. Figure 17a, c shows hydrophilicity of ZnO thin film and hydrophobicity of ZnO nanorods, respectively [256]. Water-covered endurance characteristics of devices made with nanorod layer exhibit superior switching performance as compared to device without nanorods layer, shown in Fig. 17b, d, respectively [256]. This approach not only could avoid short circuit issue due to surface wetting but also could realize water resistant electronics. Moreover, memory performance of ZnO nanorods layer based devices can be further improved by embedding in higher concentration of polymethylmethacrylate [267] or surface hydrogen annealing [261].

**Fig. 18:**
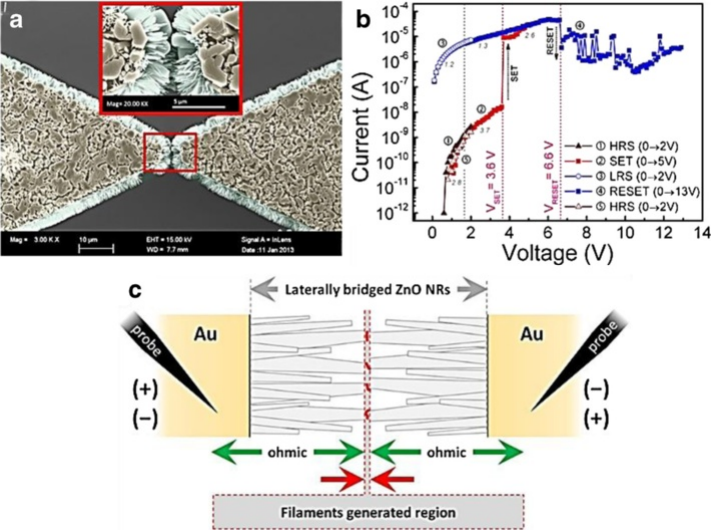
**a** Typical FE-SEM plane-view images of laterally bridged ZnO NRs. **b** Unipolar resistive switching of a laterally bridged ZnO NR-based memory device in the voltage-sweeping mode. **c** Schematic presentation of the filaments in the generated region and the ohmic conduction region [59]

Resistive memory employing laterally bridged ZnO nanorods, as shown in Fig. 18a, was developed in order to fabricate one-dimensional memory nanostructure that can meet mass production requirement [59]. Figure 18b demonstrates the peculiar unipolar switching [71] in a laterally bridged nanorod device in which the set voltage is smaller than the reset voltage that can prevent hard breakdown due to Joule heating during reset [59]. The formation and rupture of CF are situated at the nanorod/nanorod interfaces so that the actual memory cell size is incredibly smaller than the length of the nanorod itself.

**Fig. 19:**
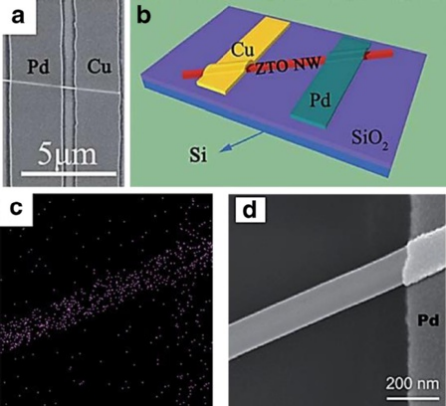
**a** SEM image of the Cu/Zn_2_SnO_4_-nanowire/Pd device. **b** a schematic view of the Cu/ZTO NW/Pd device structure. **c** EDX mapping of Cu element for the Cu/Zn_2_SnO_4_-nanowire/Pd device in the ON state. **d** The corresponding SEM image of (**c**) [74]

Another effort to downsizing the physical dimension is by developing high scalability single nanorod/nanowire resistive device [60–75]. Orthogonal crossbar or vertically aligned nanorod/nanowire array RRAM devices could greatly increase storage density due to less substrate area consumption [61, 268]. Although such arrangement has not been reported yet due to its fabrication complexity, however, the development of these one-dimensional devices offers a novel understanding in resistive switching behavior on low-scale memory devices. Figure 19a, b shows SEM image and schematic of the Cu/Zn_2_SnO_4_-nanowire/Pd device structure, respectively [74]. Energy dispersive X-ray (EDX) analysis suggests that the Cu conductive bridge is formed in Cu/Zn_2_SnO_4_-nanowire/Pd device at the surface of nanorod, as depicted in Fig. 19c, d [74]. The evidence that metal atoms originated from active anode diffused under voltage bias, which the metal atoms are mainly distributed on the surface of the nanorod, is also reported in other ZnO-nanorod-based ECM type devices [70, 73]. Similarly, oxygen diffusion toward anode on the surface of a nanorod that leads to filament formation is also reported in ZnO-nanorod-based VCM type devices [69]. This confirmed earlier hypothesis that the formation of CF occurs on the surface/sidewall rather than within the bulk of the nanorod [252, 253].

Instability and reproducibility of resistive switching in single ZnO-nanorod/nanowire can be further improved by utilizing plasma treatment [60, 61] and introduction of other metal elements, such as Cu [66], Ga, and Sb [62]. It is also reported that self-compliance and self-rectifying characteristic can be induced by Na doping on ZnO-nanowire ECM type devices [70]. Single nanorod/nanowire-based devices offer ultra-low operation current, in the range of pico- to microamperes; however, the high operation voltage, in the range of hecto- to deka-volts, is still a main challenge in the development of this kind of resistive memory device [60–62, 66, 70, 75]. Despite ZnO:K,Cl micro/nanowire devices exhibited low operation voltage; however, the operation current is in the range of milliamperes which is quite high for its class [63]. In another report, low current and voltage operation is exhibited in Ag/Zn_2_SnO_3_-sheated ZnO-core heterostructure nanowire/Ag device [67]; yet, the fabrication of this heterostructure is quite complex and may limit fabrication reproducibility.

**Fig. 20:**
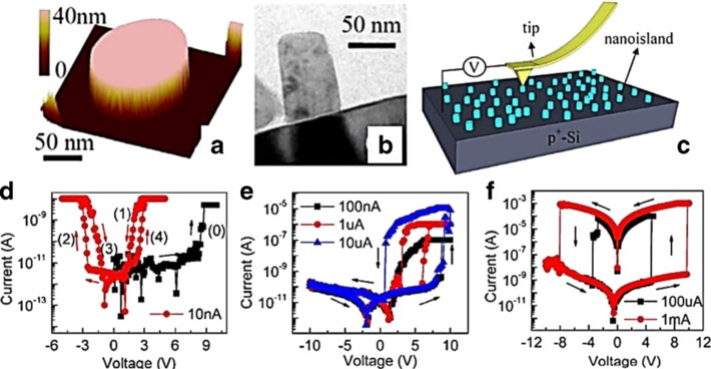
**a** Three-dimensional AFM and **b** TEM images of one nanoisland; excluding the AFM tip effect, these characterizations show that the nanoislands are discrete and having sizes between 10 and 60 nm. **c** Schematic of ZnOnanoislands and a C-AFM tip used for measurements. **d** Threshold-like, **e** self-rectifying bipolar, and **f** ordinary bipolar I-V characteristics of a ZnOnanoisland at different current compliance. The nanoisland firstly underwent process (0) as indicated in **d**, i.e., a voltage sweep from 0 to 10 V under a current compliance of 5 nA. Then, the nanoisland underwent four voltage sweep processes sequentially (process (1): 5 to 0 V; process (2): 0 to 25 V; process (3): 25 to 0 V; process (4): 0 to 5 V) as illustrated in (**d**). Under the current compliance of 10 nA, 100 nA–10 mA, and 100 mA–1 mA, the voltage sweep processes resulted in three types of resistive switching (threshold-like, self-rectifying bipolar, and ordinary bipolar) in **d**, **e**, and **f**, respectively [78]

In order to overcome the high operation voltage and current issue, the dimension of the switching device should be further scaled down. Figure 20a, b shows three-dimensional AFM and TEM images of single nanoisland grown by radio frequency plasma assisted SVTA ZnO MBE system on p^+^-Si substrate [78]. The switching behavior of the single nanoisland devices was investigated by utilizing Cr/Co coated Si tip of conducting AFM (C-AFM) as a top electrode, as depicted in Fig. 20c [78]. The device shows counterclockwise bipolar characteristic, and the range of forming, set, and reset voltage is 5.4 to 9.7 V, 1.9 to 5 V, and −1 to −4.3 V, respectively [77]. Current compliance can be set as low as 10 μA, and higher memory window can be exhibited at further increase of current compliance to 500 μA, which indicate current compliance control multilevel characteristics observed in this device [77]. Interestingly, memory window increases as the diameter of the nanoisland decreases, followed by slightly increasing of HRS resistance, yet the LRS resistance exhibited independency to the diameter [77]. The C-AFM investigation during LRS shows that the highest current is distributed at the wall of the nanoisland, which once again confirmed that the CF prefers to occur at the surface of the nanostructure [69, 77]. It is found that current compliance also may control switching mode in a single nanoisland device [78]. Threshold-like and self-rectifying characteristics exhibited when current compliance was set at 10 and 100–10 μA, respectively, after forming process, while higher than that ordinary bipolar was exhibited, as depicted in Fig. 20d–f [77, 78]. The ultra-low current compliance may control oxygen vacancies movement in the ZnO nanoisland, however, is not sufficient to form a CF [78]. Therefore, the occurrence of threshold-like and self-rectifying characteristics is due to the modulation of interfacial junction of top electrode/nanoisland and nanoisland/bottom electrode [78].

#### ZnO-Based RRAM as Multifunctional Devices

ZnO material offers a great potential in various electronic applications, such as photonic devices, spintronic devices, chemical and gas sensors, and transducers [81, 82]. It becomes more interesting when any of these properties can “coexist” with data storage applications in a single device. These multifunctional devices may revolutionize electronic circuitry, yet, still few progresses have been reported. In order to realize the multifunctional abilities, sufficient understanding in the relationship between one property and another is needed. Hence, stable and reliable multifunctional devices can be designed and fabricated appropriately.

For instance, ZnO-based transparent RRAM has a potential for being embedded in transparent wearable electronic gadget. In this case, the storage device is expected to have a stable operation in a real environment which may expose to various wavelength of light. Since ZnO is a light sensitive material that surface depletion region (SDR) may modulate the photo sensing ability; therefore, it is important to design a device that its light sensitivity should not or less affect to the resistive memory properties though it is also possible to design devices having both photonic and memory properties in the same time for certain applications.

**Fig. 21:**
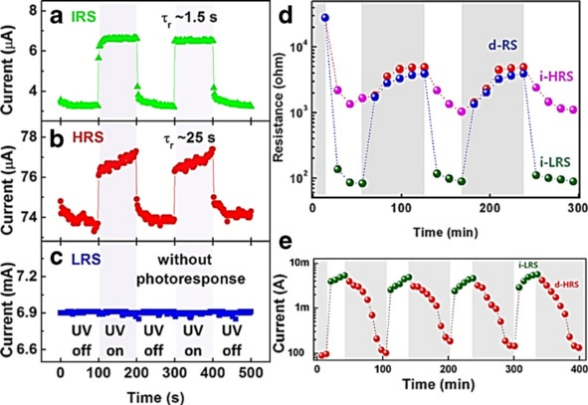
Time-resolved photocurrent under 0.5 V bias of Pt/ZnO/Pt capacitors at UV wavelength of 365 nm in the (**a**) initial resistance state (IRS), **b** high-resistance state (HRS) and **c** low-resistance state (LRS) [269]. **d** RS characteristics of Au-coated ZnOnanorods/FTO memory devices were verified by repeating the sequence of SET and RESET processes; each data was extracted at 0.01 V and (**e**) with a time to current graph by repeating the sequence of d-HRS and i-LRS under alternating illumination (at a light wavelength of 200 to 2500 nm) and dark condition cycling [255]

Recent studies found that the ultra-violet (UV) irradiation may alter resistive switching property [58, 255, 258, 265, 269, 270]. Figure 21a–c shows the time-resolved photocurrent measurements at different resistance states in Pt/ZnO/Pt memory devices [269]. Initial resistance state (IRS) and high resistance state (HRS) exhibited photo response behavior under UV irradiation, while LRS is found independent to the UV light [269]. The pronounce photocurrent in IRS and HRS is due to the suppression of SDR that can be explained by these following equations:

9 $$ {\mathrm{O}}_{2(g)}+{e}^{-}\to {\mathrm{O}}_{2\left(\mathrm{ad}\right)}^{-} $$

10 $$ {\mathrm{O}}_{2\left(\mathrm{ad}\right)}^{-}+{h}^{+}\to {\mathrm{O}}_2 $$

where ${e}^{-},{\mathrm{O}}_{2\left(\mathrm{ad}\right)}^{-}$, and *h*^+^ are single negatively charge electron, single negatively charge chemisorbed oxygen adatoms, and single positively charged hole. The chemisorbed oxygen adatoms in Eq. 9 induced SDR effect. When UV light with an energy higher than the ZnO band gap illuminates ZnO, electron-hole pairs are generated and Eq. 10 took place where the chemisorbed oxygen is discharged by photo-excited holes. The unpaired photo-excited electrons lead to photocurrent behavior in IRS and LRS; however, the metallic nature of the CF during LRS leads to independency to the light irradiation [269]. It is worth noted that the SDR need to be avoided or suppressed in ZnO-based RRAMs; it may introduce switching process instability [149, 217, 271, 272]. In addition to that, several studies suggested that resistive switching characteristics can be modulated using light irradiation treatment for several minutes [258, 270].

Interestingly, resistive switching characteristic dependent on constant light irradiation is also reported [58, 255, 265]. Figure 21d shows repetitive switching of Au-coated ZnO nanorods/FTO memory devices under dark and wide range of wavelength of light irradiation [255]. LRS and HRS can only be differentiated when the device is under irradiation. Similarly, the device can also be switched to HRS with photonic stimulus, as shown in Fig. 21e [255]. Excessive ${\mathrm{O}}_{2\left(\mathrm{ad}\right)}^{-}$ may easily recombine with oxygen vacancies regardless the applied voltage polarity, thus impeding CF formation under dark condition. Under light irradiation, the concentration of ${\mathrm{O}}_{2\left(\mathrm{ad}\right)}^{-}$ decreased due to photodesorption effect (Eq. 10) and makes CF formation possible. Good understanding in this unique relationship may allow us to expand and integrate memory and photonic application.

**Fig. 22:**
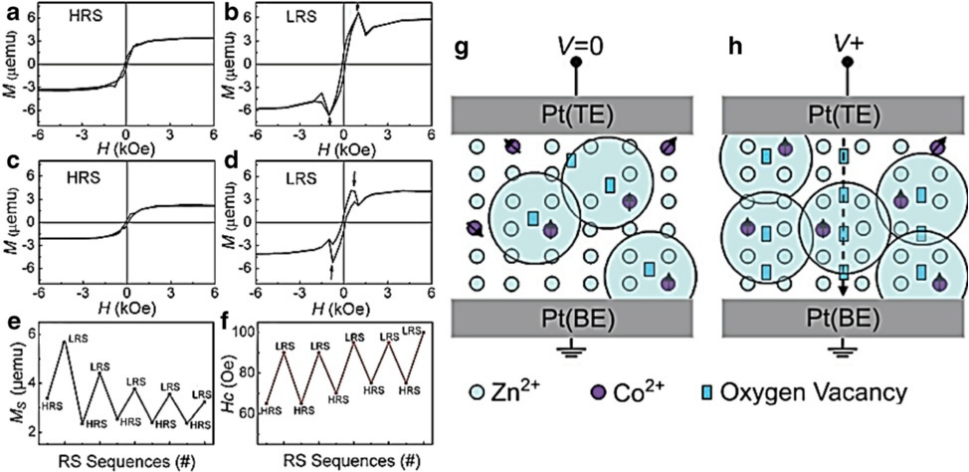
**a**-**d** Room temperature hysteresis loops of the Pt/Zn_0.95_Co_0.05_O/Pt devices for two consecutive cycles at HRS and LRS. The anomalous prostrusion in the curves indicated by arrows in **b**, **d**. The MS (**e**) and HC (**f**) are modulated reversibly by resistive switching effects. (**g**) Schematic of ferromagnetic ordering based on the BMP mechanism at HRS and (**h**) mechanism of the resistive switching and magnetic modulation during the set process [139]

ZnO material, especially Co-doped ZnO, has also attracted great attention for room temperature-diluted magnetic semiconductor (DMS) applications [273–275]. Very recently, it is found that resistive switching characteristics have a direct correlation with magnetic modulation (MM) [139]. Figure 22a–f shows that resistive switching induced ferromagnetism in Pt/Co:ZnO/Pt devices [139]. The magnetic behavior can be easily tuned by simply changing the resistance state. The result in Fig. 22 shows that both saturation magnetization (Ms) and coercive field (Hc) are more pronounced in LRS [139]. This phenomenon can be explained using bound magnetic polarons (BMPs) model. Higher amount of oxygen vacancies created during LRS leads to higher volume occupied by BMPs; therefore, more Co^2+^ ions were overlapped into the ferromagnetic domains; conversely, the oxygen vacancy annihilation in HRS results in the decreasing of magnetic ordering, as presented in Fig. 22g–h [139]. Thus, this finding may encourage further development of multi-state data storage employing both electrical and magnetic properties.

Further exploration on the influence of light irradiation/exposure and magnetic modulation to the reliability and stability of switching characteristics are needed. Related to that, those reported studies only focus on VCM devices, while, based on our literature study, studies on multifunctional behavior on ECM devices are still less discussed. By employing the strain-induced polarization charges produced at the semiconductor/metal interface under externally applied deformation as a result of piezotronic effect, the switching characteristics of the CVD grown ZnO NW (diameter 500 nm; length 50 μm) resistive switching devices on PET are also reported [276]. In addition, the correlation between switching properties and other unique properties of ZnO such as lateral photovoltaic effect [277], electroluminescence [278, 279], piezoelectricity [276, 280, 281], light emitters [282] are still not yet fully explored.

Apart from its potential as a multifunctional RRAM material, ZnO performance among other oxides-based is also quite comparative. Table 6 shows performance comparison between different metal oxides in published literature. ZnO may offer sufficient endurance and memory window with acceptable retention performance; yet, much lower operation current and faster operation speed using other oxides are reported. This indicates that low current operation and tolerable operation speed are a great challenge for the development of ZnO-based RRAM. In addition, the development of ultra-thin ZnO-based RRAM is needed, since utilization of switching layer is expected to scale down to 1× nm in the near future [283].

**Table 6:** Performance comparison of Pt or Ag/metal oxide/Pt in published literature

No	Structure	SL thickness (nm)	Maximum operation current (mA)	Set/reset operation speed (μs)	Endurance/ratio (cycles)/(times)	Retention (seconds or hours)	Stress (seconds)	Ref.
1	Pt/ZnO/Pt	25	3	10^4^	10^6^(AC)/>10^2^	>6 × 10^5^s/RT	NA	[122]
2	Pt/Al_2_O_3_/Pt	2	~1	0.07	10^5^/10^2^	NA	NA	[289]
3	Pt/NiO/Pt	10	0.1	0.1/~1	100/~10	1000 h/150 °C	NA	[290]
4	Pt/TaOx/Pt	30	<0.17	0.01	10^9^/~10	3000 h/150 °C	NA	[291]
5	Pt/TiO_2_/Pt	27	3	DC	80/~10^2^	NA	NA	[292]
6	Pt/ZrO_2_/Pt	130	~10	10^4^/5 × 10^4^	10^5^/10^3^	NA	NA	[293]
7	Pt/Gd_2_O_3_/Pt	120	35	DC	60/>10^6^	30 h/85 °C	NA	[294]
8	Ag/ZnO/Pt	100	10	DC	40/10^2^	NA	10^4^/RT	[124]
9	Ag/La_2_O_3_/Pt	50	0.035	DC	>10^3^/>10^3^	10^6^ s/RT	NA	[295]
10	Ag/SiO_2_/Pt	80	0.5	DC	~35/10^6^	~2 × 10^3^s/RT	NA	[296]
11	Ag/TaO_x_/Pt	65	~100	NA	NA	NA	NA	[297]
12	Ag/ZrO_2_/Pt	50	5	DC	>10^2^/>10^2^	NA	~7.5 × 10^3^/RT	[298]
13	Ag/TiO_2_/Pt	40	0.29	100/10^3^	~10/~10^6^	10^4^ s/RT	NA	[299]

*RT* measured at room temperature, *NA* data not available, *DC* direct-current voltage sweeping mode

## Conclusions

In the case of VCM cell, the area of switching region can be controlled by modulating the microstructural properties and defects concentration of ZnO films; however, in ECM cell, relying only on those factors are insufficient; another technique, probably by electrode engineering approach, needs to be developed in order to control the Joule heating effective region.

Both VCM and ECM cells require high resistivity of ZnO films in order to achieve suitable memory effect. Though several efforts have been conducted to suppress the native defects concentration to achieve less leakage current, yet development of p-type and superoxide ZnO-based RRAM has not been explored yet. The development of highly resistive ZnO film may open the possibility to thinning down the switching layer and lowering the current operation; fabrication of ultra-thin and low power device is the major challenge in this oxide system. ZnO nanoisland-based switching memory device is a promising approach for the low power scalable memory devices.

It is also quite interesting and challenging at the same time to explore the multifunctional RRAM. Up to now, most reports on these correlation studies are still only in the early stage. We believe that investigation and development of multifunctional nonvolatile memory devices will attract significant interest in the near future.
